# Shared genetic architecture of psychiatric disorders and hemorrhoidal disease: a large-scale genome-wide cross-trait analysis

**DOI:** 10.3389/fpsyt.2024.1456182

**Published:** 2024-11-11

**Authors:** Zhangsendi Chen, Bowen Hu, Ji Sun, Yuhong Jiang, Zhe Chen, Chunmei Yang, Hongbo He, Weiguo Wang

**Affiliations:** ^1^ Division of Surgery, Institute of Integrated Traditional Chinese and Western Medicine, West China Hospital, Sichuan University, Chengdu, Sichuan, China; ^2^ Department of Integrated Traditional Chinese and Western Medicine, National Clinical Research Center for Geriatrics, West China Hospital, Sichuan University, Chengdu, China; ^3^ Department of Thoracic Surgery, The Second Xiangya Hospital, Central South University, Changsha, Hunan, China

**Keywords:** hemorrhoidal disease, psychiatric disorders, genetic overlap, pleiotropic loci, Mendelian randomization

## Abstract

**Background:**

The genetic association between psychiatric disorders and hemorrhoidal disease (HEM) is still not well known. The work aims to investigate their comorbidity at a genetic level.

**Methods:**

Utilizing recent large-scale genome-wide association studies (GWAS), we investigated the genetic overlap at the single nucleotide polymorphism (SNP), gene, and molecular level between depression and HEM, bipolar disorder (BD) and HEM, neuroticism and HEM, as well as schizophrenia (SCZ) and HEM. The cross-trait genes were validated through the utilization of transcriptome and proteome methodologies. The causal link was assessed using bidirectional two-sample Mendelian randomization analysis (MR) analysis. MRlap corrects for the potential bias in estimation caused by sample overlap.

**Results:**

We discovered significant positive genetic associations between these four types of psychiatric disorders and HEM. Cross-phenotypic association analyses identified shared SNPs along with 17 specific loci between psychiatric disorders and HEM. MAGMA identified a total of 2304 pleiotropic genes, several of which showed significant expression in the results of transcriptome and proteome analyses. We observed that these genes are mostly associated with the regulation of transcription factors and particular DNA binding activities. Lastly, MR analysis provided evidence supporting a correlation between these conditions.

**Conclusion:**

This study revealed a genetic correlation between four psychiatric disorders and HEM, identified pleiotropic loci, found multiple candidate genes, and confirmed causal relationships. This has enhanced our comprehension of the common genetic mechanisms of psychiatric disorders and HEM.

## 1 Introduction

Hemorrhoidal disease (HEM) is a prevalent anorectal condition characterized by symptomatic enlargement and distal displacement of the normal anal cushions (1). The psychiatric concerns of patients with HEM have been consistently present in clinical practice, yet are frequently disregarded. The symptoms of pain, bleeding, itching, swelling, and other discomforts associated with HEM can have a substantial influence on a patient’s daily life and reduce their overall quality of life (2). Patients suffering from this private ailment may accumulate distressing stress over an extended period (3), which can potentially trigger psychiatric problems. Observational studies indicate that psychological and psychiatric factors may play a crucial role in hemorrhoid development (4). Akkoca et al.’s study found that the levels of psychological symptoms and aggression among patients with HEM were significantly higher than those in the control group (5). A national health and nutrition examination survey I dentified depression as a risk factor for HEM (6). The coexistence of psychiatric disorders and HEM has emerged as a significant global challenge (7). Nevertheless, the connection between psychiatric disorders and HEM cannot be conclusively proven due to inconsistencies in research methodology, the diversity of populations studied, and the inherent limits of observational settings, including the possibility of reverse causality effects and environmental factors that may influence the results. In order to further examine the cause-and-effect relationship between the two variables, it is necessary to utilize instrumental factors such as genetic variations.

Large-scale genome-wide association studies (GWAS) have made substantial progress in identifying genetic variations linked to psychiatric disorders and HEM. Through a comprehensive GWAS analysis, 102 independent risk loci for HEM were identified, revealing significant associations with neuroaffective disorders (8). This could be mediated by similar mechanisms involving intestinal motility to mediate genetic risk effects. However, our understanding of the common etiology and genetic susceptibility underlying both psychiatric disorders and HEM remains incomplete. To address this gap, a Mendelian randomization (MR) study was conducted to assess the causal relationship between psychiatric disorders and HEM (9). While MR addresses confounding factors and reverse causality, it does not account for pleiotropy, where genetic variants are associated with multiple traits simultaneously (10, 11). Therefore, more advanced approaches are needed to comprehensively understand the association between psychiatric disorders and HEM as well as elucidate the underlying biological mechanisms in order to facilitate effective prevention and management of this comorbid condition.

This study utilized GWAS summary data at both the SNP and gene levels to conduct a comprehensive pleiotropic analysis of four major psychiatric disorders (depression, bipolar disorder, neuroticism, and schizophrenia) and HEM, aiming to systematically investigate the genetic correlation, shared genetic loci and genes. Bidirectional two-sample MR analysis was employed to explore the causal components of the genetic association. A multifaceted approach was adopted for genome-wide cross-trait analysis.

## 2 Methods

### 2.1 GWAS datasets

**Figure 1:**
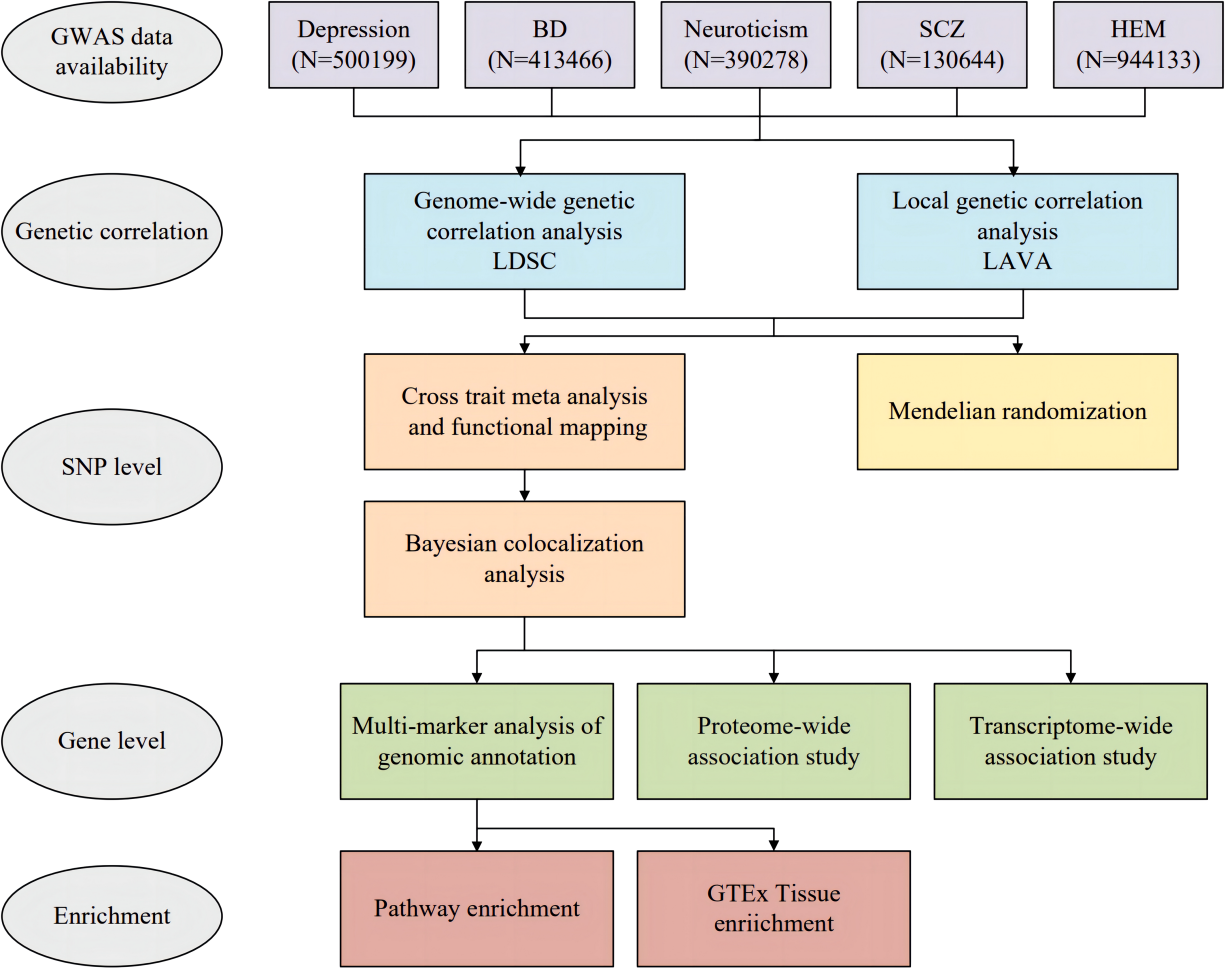
Overall study design of genome-wide cross-trait analysis. BD, bipolar disorder; SCZ, schizophrenia; HEM, hemorrhoidal disease.

The overall study design is depicted in **Figure 1**. Participants were selected from European descent public data sets for the study. Summary statistics on HEM were obtained through a meta-analysis of GWAS datasets, which included a total of 944,133 subjects (8). We acquired GWAS summary data for depression from a meta-analysis that encompassed 807,553 subjects and combined the three largest available depression genetic studies (UK Biobank, 23andMe, and Psychiatric Genomics Consortium) (12). Due to the availability of data from 23andMe, we obtained summary data for approximately 500199 individuals (170756 cases and 329443 controls). Additionally, we acquired GWAS summary data for bipolar disorder (BD) and schizophrenia (SCZ) from the Psychiatric Genomics Consortium (PGG) (https://pgc.unc.edu/for-researchers/download-results/). The data set of neuroticism was included as an additional component. While neuroticism is not officially classified as a psychiatric disorder, it is recognized as a personality trait closely associated with psychiatric conditions such as depression and anxiety. Its genetic architecture has demonstrated significant overlap with common psychiatric disorders (13). The GWAS summary data for Neuroticism were obtained from the Center for Neurogenomics and Cognitive (CNCR) (14). Specifically, **Supplementary Table S1** provides detailed information about each GWAS study. We excluded SNPs with duplicate or missing rsID numbers from the dataset and mapped the chromosomal positions of all SNPs to the hg19 human reference genome. For quality control purposes, we retained only SNPs in the 1000 Genomes European population (15).

### 2.2 General genetic correlation analysis

We employed linkage disequilibrium (LD) (LDSC; https://github.com/bulik/ldsc) score regression (16) and high-definition likelihood (HDL) (17) methods to examine the overall genetic association between four psychiatric disorders and HEM. The LDSC method recognizes that the estimated effect size of a particular SNP reflects not only its own impact but also the combined effects of all other SNPs in LD with it. Genetic correlation between different traits can be estimated using GWAS summary statistics by considering this relationship (18). Univariate LDSC was initially utilized to estimate the heritability (h2) of individual traits, while bivariate LDSC with unconstrained intercepts calculated the genetic correlation (rg) and genetic covariance for the four psychiatric disorders and HEM, respectively. The range of genetic correlation estimates is from -1 to +1. LD Scores were estimated from European ancestry samples in the 1000 Genomes Project (18). As an extension of the LDSC approach, The HDL method naturally expands upon the regression formula of LDSC and provides a more precise estimation of genetic correlation (17).

### 2.3 Local genetic correlation analysis

To tackle the issue that conventional global approaches may fail to detect signals localized in specific regions or in opposing directions at different loci, the local analysis of covariant association (LAVA) method was supplemented with (19). Using the 1000 Genomes Project’s European panel as a reference for linkage disequilibrium (LD), we stratified the GWAS dataset into 2495 loci and used multivariate genetic association analysis to identify common genetic association regions and conditional local genetic relationships between the four psychiatric disorders and HEM.

### 2.4 Cross-trait meta-analysis

To investigate the genetic pleiotropy of a specific locus on two distinct traits, we employed a comprehensive cross-phenotype association analysis (CPASSOC) at the genome-wide level to identify shared loci between psychiatric disorders and HEM. As one of the statistical methods utilized in CPASSOC, unlike Shom, which is a linear combination of univariate test statistics, SHet statistics can be effectively approximated by a shift gamma distribution that accommodates heterogeneous effects resulting from different studies on diverse traits (20).

### 2.5 Genomic loci characterization and functional annotation

The SNP2GENE method of Functional mapping and annotation (FUMA) platform was employed for functional mapping and annotation to further ascertain independent genomic loci (21). SNPs meeting the criteria of P_CPASSOC_<5×10−8 and LD r2<0.6 were defined as independent significant SNPs, while the lead SNP was determined based on its independence significance and LD r2<0.1. Genomic loci closer than a distance of 500 kb were merged into a single locus, with reference panel data from the European population of the 1000 Genomes Project Phase 3 being applied. For each single trait, we established a threshold of P_single-trait_<5e-08 to obtain its respective genomic loci, which were then compared against those obtained from previous meta-analyses to identify novel pleiotropic. ANNOVAR provided functional information for SNPs located within genes. The CADD score in the annotated information was used as the harm score, which below 12.37 was considered indicative of reduced potential adverse protein effects.

### 2.6 Bayesian colocalization analysis

Bayesian colocalization analyses (COLOC) (22) were conducted using the combined SNPs from two GWAS in each study group to assess the likelihood of shared genetic causal variants between two traits by exploring potential pleiotropic loci. The COLOC analysis was based on five exclusive posterior probabilities: H0 (no association with either trait), H1 (genetic association only with trait 1), H2 (genetic association only with trait 2), H3 (association with both traits but independent SNPs), and H4 (association with both traits and only one shared SNP). Co-localized loci in cross-trait analysis were identified as those having PP4 > 0.70.

### 2.7 Multi-marker analysis of GenoMic Annotation

Gene and gene set analyses have been identified as potentially more effective alternatives to SNP analysis. In gene analysis, genetic marker data are aggregated at the level of the entire gene to assess the collective association of all markers in the gene with the phenotype. Gene set analysis can also provide additional insights into the functional and biological mechanisms underlying the genetic components of traits. Multi-marker Analysis of GenoMic Annotation (MAGMA) is a rapid and flexible method for conducting gene and gene-set analyses within a two-tiered parametric framework (23). MAGMA’s gene analysis utilizes a multiple regression approach that effectively combines LD between markers and identifies multi-marker effects, demonstrating superior computational efficiency compared to alternative tools. We obtained overlapping loci by aligning coding genes’ positions in NCBI build 37.3 with all pleiotropy loci identified by CPASSOC. Subsequently, MAGMA was utilized to perform association analysis on candidate overlapping pleiotropy genes. The gene set generated by MAGMA was intersected with both FUMA’s physically annotated gene set and MAGMA’s own generated gene set, resulting in the identification of pleiotropic genes at the individual level after BH correction.

### 2.8 Transcriptome-wide association studies

The utilization of diverse methodologies for identifying overlapping genes can mitigate the potential for errors and enhance the elucidation of causal mechanisms underlying interrelated traits. We used GTEx (Genotype-Tissue Expression, version 8) transcriptome data from 16 different regions of brain tissue, sigmoid colon, and whole blood as a reference panel to perform transcriptome-wide association studies (TWAS) using FUSION software (24). The results of single-trait TWAS were then combined to identify gene-tissue pairs that are shared among the four psychiatric disorders and HEM.

### 2.9 Proteome-wide association studies

The enhanced comprehension of genetic regulation in the proteome facilitates the identification of causal mechanisms underlying complex traits. In comparison to trans-associations, cis-associations exhibit greater reproducibility across diverse proteomic platforms (25). Proteome-wide association studies (PWAS) were performed to identify cross-trait protein expression using plasma protein cis-pQTL data from individuals of European ancestry.

### 2.10 Biological pathway and tissue enrichment analysis

We evaluated the enrichment of overlapping genes between CPASSOC and MAGMA in gene Ontology (GO) (26) biological processes and Kyoto Encyclopedia of Genes and Genomes (KEGG) (27) pathways using MAGMA Gene-set analysis (23) to better understand the biological significance of genes across traits. Gene sets corresponding to colocalized loci were focused on for further exploration. In addition, the FUMA analysis platform was utilized to perform tissue enrichment analysis on 53 tissue types provided by GTEx v.8, enabling the identification of tissues associated with shared genes (21).

### 2.11 Mendelian randomization

We conducted a comprehensive Bidirectional two-sample MR analysis of the relationship between HEM and depression, BD, neuroticism, and SCZ. We applied a genome-wide association significance threshold of P < 5.0 × 10-8, excluded single nucleotide polymorphisms (SNPs) with palindromic SNPs and linkage disequilibrium structures (r2 < 0.001 within 10000 kb), and removed weak instrumental variables with an F-statistic less than 10. To ensure consistent SNP effects, we harmonized the exposure and outcome data to account for allele differences. Given the heterogeneity among SNPs, we primarily employed random effects inverse variance weighting (IVW) in our MR analysis (28), supplemented by MR-Egger regression to detect potential bias caused by directional pleiotropy (29). Due to the possibility of sample overlap in the summary data from European populations, as well as the possibility of sample overlap in the pooled UK Biobank (UKB) data for depression and HEM, we applied MRlap to correct for the estimated bias in the IVW results. MRlap uses cross-feature LDSC to approximate overlap, allowing it to evaluate and correct for the bias introduced by sample overlap in MR analyses (30). We assessed heterogeneity using the MR-Egger intercept test and Cochran’s Q test. Additionally, we utilized MR-Egger regression as well as MR-pleiotropy residual and outlier (MR-PRESSO) methods to identify and address any potential horizontal pleiotropy (31).

## 3 Results

### 3.1 Genetic correlations

Univariate LDSC showed a significant genetic effect for each trait. After adjusting for multiple tests, significant genetic associations were identified between HEM and all four psychiatric disorders. Depression exhibited the most pronounced genetic effect (rg = 0.28, P = 2.04E-34), followed by notable positive associations observed for BD (rg = 0.142, P = 1.42E-10), neuroticism (rg = 0.197, P = 1.55E-20), and SCZ (rg = 0.101, P = 6.58E-07). The HDL model we utilized further validated the presence of a significant positive correlation (**Table 1**). The results of local genetic correlation analysis of LAVA revealed significant local genetic correlations between psychiatric disorders and HEM. Following FDR multiple corrections, we identified 49 specific associations between depression and HEM, with the most prominent position at chr3: 113657666-115649909(P = 4.38E-07, rho = 0.59). Additionally, we observed twenty-four distinct relationships between BD and HEM, forty-eight connections between neuroticism and HEM, as well as twenty-four links between SCZ and HEM (**Supplementary Table S2–S5**).

**Table 1:** Genome-wide genetic correlation between psychiatric disorders and HEM by LDSC and HDL.

Method	Trait1	h2_trait1_ (SE)	Trait2	h2_trait2 _(SE)	rg (95%CI)	*P*-value	Intercept (SE)
LDSC	Depression	0.06 (0.0024)	HEM	0.029 (0.0012)	0.28 (0.2347,0.3245)	2.04E-34	0.016 (0.0064)
	BD	0.071 (0.0027)			0.142 (0.0988,0.1854)	1.42E-10	0.004 (0.0062)
	Neuroticism	0.102 (0.0035)			0.197 (0.1552,0.2384)	1.55E-20	0.014 (0.0068)
	SCZ	0.359 (0.0115)			0.101 (0.0611,0.1403)	6.58E-07	0.008 (0.0066)
HDL	Depression	0.05(0.0018)	HEM	0.025 (0.0009)	0.27 (0.2221,0.3173)	1.14E-28	0.009 (0.0007)
	BD	0.059 (0.0020)			0.142 (0.1053,0.1779)	2.03E-14	0.005 (0.0007)
	Neuroticism	0.074 (0.0023)			0.194 (0.1566,0.2320)	4.84E-24	0.008 (0.0008)
	SCZ	0.301 (0.0087)			0.122 (0.0926,0.1510)	3.24E-16	0.011 (0.0013)

h2, heritability; rg, genetic correlation; SE, standard error; HEM, hemorrhoidal disease; BD, bipolar disorder; SCZ, schizophrenia.

### 3.2 Cross-trait meta-analysis and SNP annotation

**Figure 2:**
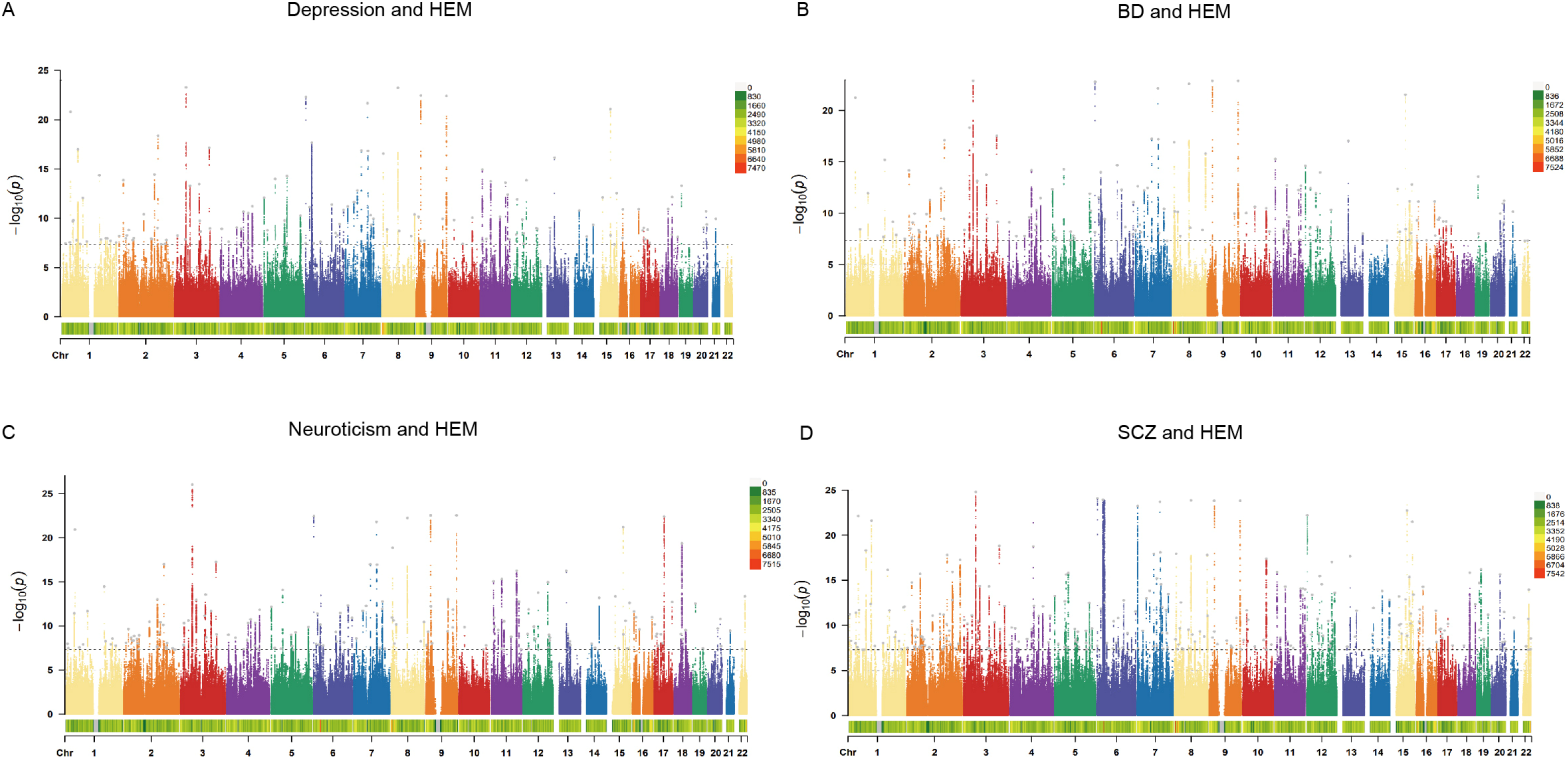
The Manhattan plots of CPASSOC. The x-axis represents the chromosomal location of the SNPs, and the y-axis represents the significance (-log10P). The bottom of the Manhattan plots represents the chromosome density. The gray dots represent the independent pleiotropic loci annotated by FUMA. **(A)** The Manhattan plot of shared SNPs based on results of CPASSOC between depression and HEM. **(B)** The Manhattan plot of shared SNPs based on results of CPASSOC between BD and HEM. **(C)** The Manhattan plot of shared SNPs based on results of CPASSOC between neuroticism and HEM. **(D)** The Manhattan plot of shared SNPs based on results of CPASSOC between SCZ and HEM. HEM: hemorrhoidal disease, BD: bipolar disorder, SCZ: schizophrenia.

The CPASSOC analysis identified SNPs significantly associated with at least one psychiatric disorder or HEM in each cohort through four genome-wide meta-analyses. Using FUMA, we further annotated the results and discovered independent pleiotropic loci, including 134 loci shared between depression and HEM, 140 loci shared between BD and HEM, 170 loci shared between neuroticism and HEM, as well as 266 loci shared between SCZ and HEM (**Figure 2**; **Supplementary Table S6–S9**). Multiple loci expressed significance across all pairwise traits, indicating broad pleiotropy at these loci, such as Index SNP rs9847710 (mapped gene: SFMBT1:RP11-894J14.5), Index SNP rs55646585 (mapped gene: PLEC), and Index SNP rs12474027 (mapped gene: MYT1L). Interestingly, the index SNP rs4910165 mapped in MRVI1 and rs6498573 mapped in MYH11, which showed significance in all four groups of analyses, were related to extracellular matrix organization and muscle function and have been elucidated to be associated with HEM (6). The MYH11 encodes a smooth muscle myosin that is involved in muscle contraction, relaxation and gastrointestinal motility disorders and shows increased expression in HEM transcriptome analysis. Non-coding variants in MYH11 have also been found to be associated with dementia in women with differential expression in microarray study of frontal cortex (32). The Index SNP rs2186797, located at chr11:69971277-70028543 and mapped in ANO1, exhibited significant expression in the meta-analysis of SCZ and HEM. It demonstrated a CADD score of 27.6, which was the highest among all pairwise traits. After ANO1 activation, there is an efflux of chloride ions, resulting in cell depolarization and elevated membrane potential. This leads to the activation of calcium channels and enhanced cell contraction, which are essential for normal gastrointestinal peristalsis and have also been implicated in the pathogenesis of HEM (8, 33). Variants in non-coding regions accounted for the highest proportion of identified SNPS, and a summary of SNP annotations for each pair of traits is shown in **Supplementary Figure S1**.

Known pleiotropic loci overlapping in each trait pair were obtained by alignment with FUMA annotation results from single-trait GWAS (P<5e-08). In addition, SNPS that did not overlap with the significant loci of the two single-trait GWAS were compared with the P values of the two single-trait GWAS, and 5× 10-08 <P<1× 10-03 loci were considered as novel pleiotropic loci of greatest interest. Finally, we identified 21 of the most important novel pleiotropic loci (**Supplementary Table S10**), including 9 pleiotropic loci between depression and HEM, 6 pleiotropic loci between BD and HEM, 3 pleiotropic loci between neuroticism and HEM, and 3 novel pleiotropic loci between SCZ and HEM. The most significant novel pleiotropic locus (Index SNP: rs12705959, P_CPASSOC_ = 4.95E-11) between depression and HEM was located near the intron of FOXP2. The expression of FOXP2 in brain tissue is significantly elevated (34), indicating its association with genetic speech and language disorders, and its involvement in regulating motor function (35). For BD and HEM, a novel pleiotropic topSNP rs144767533 was identified, which was located near the non-coding RNA intron of CUL9. It was also the mapped gene for the most significant novel locus between neuroticism and HEM.

### 3.3 Colocalization analysis

A colocalization analysis was conducted to determine whether the multi-effective SNPs driving the association between the two traits are the same. The COLOC analysis identified shared genetic causal variants between each pair of traits in the four sets of CPASSOC outcomes for psychiatric disorders and HEM (**Table 2**). A total of 17 loci were identified in the four analyses. Among the 134 significant pleiotropy loci associated with depression and HEM, three loci exhibited a PP4 value exceeding 0.7, indicating a potential shared genetic basis underlying these two traits. The significant locus with the highest posterior probability of association between depression and HEM (PPH4 = 88.46%, mapped gene: CELF4) was located in the intergenic region on chromosome 18. It is worth noting that locus 17 (PPH4 = 82.04%, mapped gene: MYT1L) was also identified as one of the most important novel pleiotropic loci in the previous CPASSOC analysis of depression and HEM. The most significant locus 137 (PPH4 = 96.03%) between BD and HEM was mapped to the intergenic region of OSBPL2 on chromosome 20, which plays a key role in lipid transport (36). Three loci overlapped with previous novel pleiotropy loci between BD and HEM. In addition, locus 122 (PPH4 = 71.46%) between SCZ and HEM also coincided with a novel pleiotropic locus in this group.

**Table 2:** Results of colocalization analysis of pleiotropic loci between psychiatric disorders and HEM identified from CPASSOC.

GenomicLocus	TopSNP	Chr: position	A1	A2	BETA		*P*-value		*P*_CPASSOC_	PP4	NearestGene
					Trait	HEM	Trait	HEM			
Depression and HEM											
126	rs1557339	18:35129076	A	C	0.029	0.024	4.37E-09	2.98E-07	1.14E-11	0.884580042	CELF4
95	rs10838738	11:47663049	A	G	-0.021	0.025	2.99E-06	1.69E-09	1.76E-14	0.82780693	MTCH2
17	rs55933406	2:2297348	C	G	0.021	0.02	1.07E-05	2.35E-06	6.31E-09	0.820422933	MYT1L
BD and HEM											
137	rs13044225	20:60865815	A	G	0.055	-0.02	8.50E-09	8.34E-07	6.08E-12	0.960301673	OSBPL2
132	rs62109878	19:13105333	C	G	-0.046	-0.018	2.39E-06	9.47E-06	9.43E-09	0.90269178	NFIX
36	rs2007403	4:106131210	C	T	0.039	0.028	4.53E-05	1.10E-11	6.57E-15	0.84862958	TET2
55	rs144767533	6:43186138	C	T	-0.062	0.032	4.67E-05	3.52E-07	3.70E-10	0.840720864	CUL9
49	rs12153515	5:164631794	C	T	0.055	0.037	6.99E-05	7.33E-10	1.26E-12	0.817408791	CTB-181F24.1
82	rs55646585	8:144999621	C	T	0.058	0.027	6.95E-09	5.10E-11	1.58E-16	0.781187052	PLEC
115	rs12908161	15:85207825	A	G	-0.065	-0.022	4.68E-10	6.40E-07	6.61E-12	0.711169399	SEC11A
10	rs11684360	2:26942156	C	T	0.042	0.025	3.09E-04	2.68E-07	3.27E-09	0.70348687	KCNK3
Neuroticism and HEM											
46	rs827186	3:158047235	C	T	-0.023	-0.033	1.31E-07	1.39E-05	2.48E-08	0.854796741	RSRC1
77	rs7749650	6:152044872	A	T	0.012	-0.026	4.27E-06	6.80E-09	4.76E-13	0.836922977	ESR1
SCZ and HEM											
116	rs144767533	6:43186138	C	T	-0.065	0.032	3.62E-06	3.52E-07	8.91E-09	0.919688426	CUL9
23	rs6715366	2:2327295	A	G	0.054	0.018	2.49E-08	3.17E-05	3.10E-08	0.84936598	MYT1L
228	rs11638554	15:85148231	G	T	-0.065	-0.02	7.58E-12	3.28E-06	5.10E-12	0.786392303	ZSCAN2
122	rs12207616	6:111608797	A	T	0.055	0.029	1.45E-05	2.78E-07	2.93E-08	0.714630982	RP5-1112D6.4

A1, effect allele; A2, alternative allele; HEM, hemorrhoidal disease; BD, bipolar disorder; SCZ, schizophrenia; PP4, the posterior probabilities of these two features are correlated and share a single causal variant; NearestGene, the nearest gene of the SNP based on ANNOVAR annotations.

### 3.4 Gene-level analysis

A total of 2304 pleiotropic genes with significant associations were identified by MAGMA across the four pairs of traits after correction (FDR qvalue < 0.05). These genes overlapped with the shared loci identified by CPASSOC, including 416 genes between depression and HEM, 495 genes between BD and HEM, 487 genes between neuroticism and HEM, and 906 genes between SCZ and HEM (**Supplementary Table S11-S14**). Secondly, we identified 17 candidate genes associated with both depression and HEM by matching the aforementioned genes to previously identified colocalization regions. Additionally, we discovered 25 candidate genes shared between BD and HEM, 6 candidate genes between neuroticism and HEM, as well as 18 candidate genes between SCZ and HEM (**Supplementary Table S15**). MTCH2 (P adjust = 1.08E-11) was the most significant gene between depression and HEM, and it was also a mapped gene for one of the shared loci identified in the previous colocalization analysis between depression and HEM. In addition, the significant gene MYT1L (P adjust = 4.55E-4) was also consistent with the FUMA annotation results based on CPASSOC analysis. For BD and HEM, several significant genes, such as PLEC (P adjust = 5.25E-13) on chromosome 8 overlapped with mapped genes of colocalized genetic loci. ESR1 (P adjust = 1.11E-12) was the most significant gene between neuroticism and HEM. After correction, significant SNP-heritability enrichment across multiple organizations was identified, mainly enriching in various brain tissues. The cerebellum exhibited significant enrichment in all four pairs of traits (**Supplementary Figure S2**). We further used FUSION for TWAS based on the significant genes identified by MAGMA to investigate the effects of gene expression levels and tissue specificity. We also conducted PWAS to identify plasma protein-trait associations.

**Figure 3:**
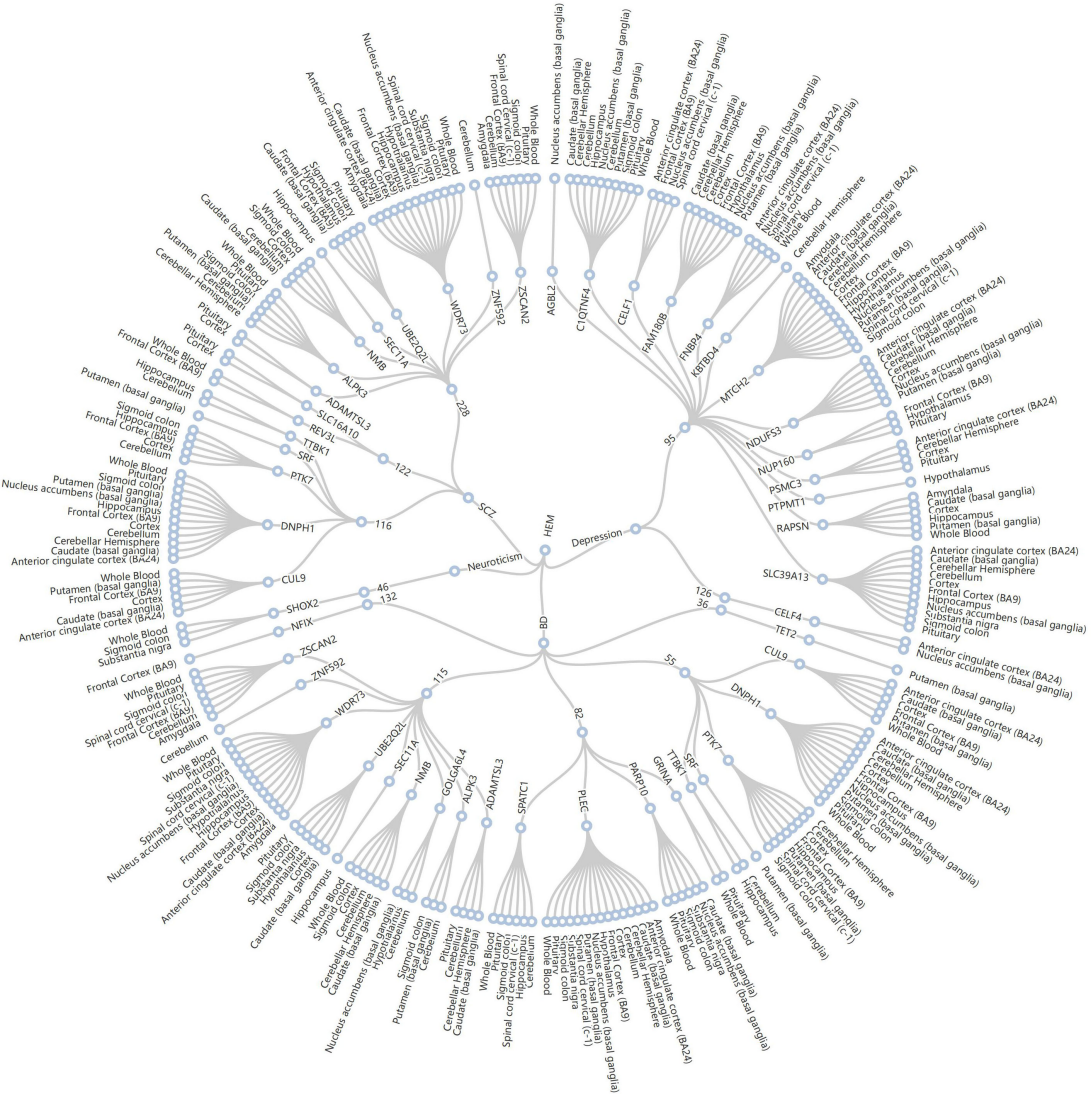
Radial dendrogram of TWAS based on colocalized regions. 11 loci for HEM (center point) and four psychiatric disorders (inner circle) fell within the colocalization region (circle 2). The 50 shared genes involved (circle 3) were shared in 16 tissues (circle 4). HEM, hemorrhoidal disease; BD, bipolar disorder; SCZ, schizophrenia.

We obtained the set of tissue-gene pairs shared by each trait after tissue-specific FDR correction. There were 440 shared gene-tissue pairs observed between depression and HEM (with 109 shared genes), 436 shared gene-tissue pairs observed between BD and HEM (with 108 shared genes), 460 shared gene-tissue pairs observed between neuroticism and HEM (with 122 shared genes), and 763 shared gene-tissue pairs observed between SCZ and HEM (with 219 shared genes) (**Supplementary Tables S16–19**). A total of 11 loci fell within the colocalization analysis (**Figure 3**). Of the 14 genes shared between depression and HEM, MTCH2 was shared simultaneously by 2 traits in 13 of the tissues. Two traits share PLEC in 15 of the tissues among the 20 genes between BD and HEM. There are several shared genes that overlap with previously MAGMA co-localized regions, such as DNPH1, ZSCAN2, ALPK3, CUL9, and PTK7 between SCZ and HEM. Based on the significant genes identified by MAGMA, PWAS identified a total of 86 plasma proteins as significantly associated with four traits after multiple testing corrections (FDR qvalue< 0.05) (**Supplementary Table S20**). There are four plasma proteins shared between depression and HEM, six plasma proteins shared between BD and HEM, two plasma proteins shared between neuroticism and HEM, and seven plasma proteins shared between SCZ and HEM. All pairwise traits share two protein-coding genes (ITIH1, ITIH3). Comparing the shared genes in TWAS of whole blood with the shared proteins in PWAS, we found that the protein-coding gene ITIH4 reached significant levels of mRNA and protein in three pairs of traits of depression, BD and SCZ.

### 3.5 Functional enrichment analysis

**Figure 4:**
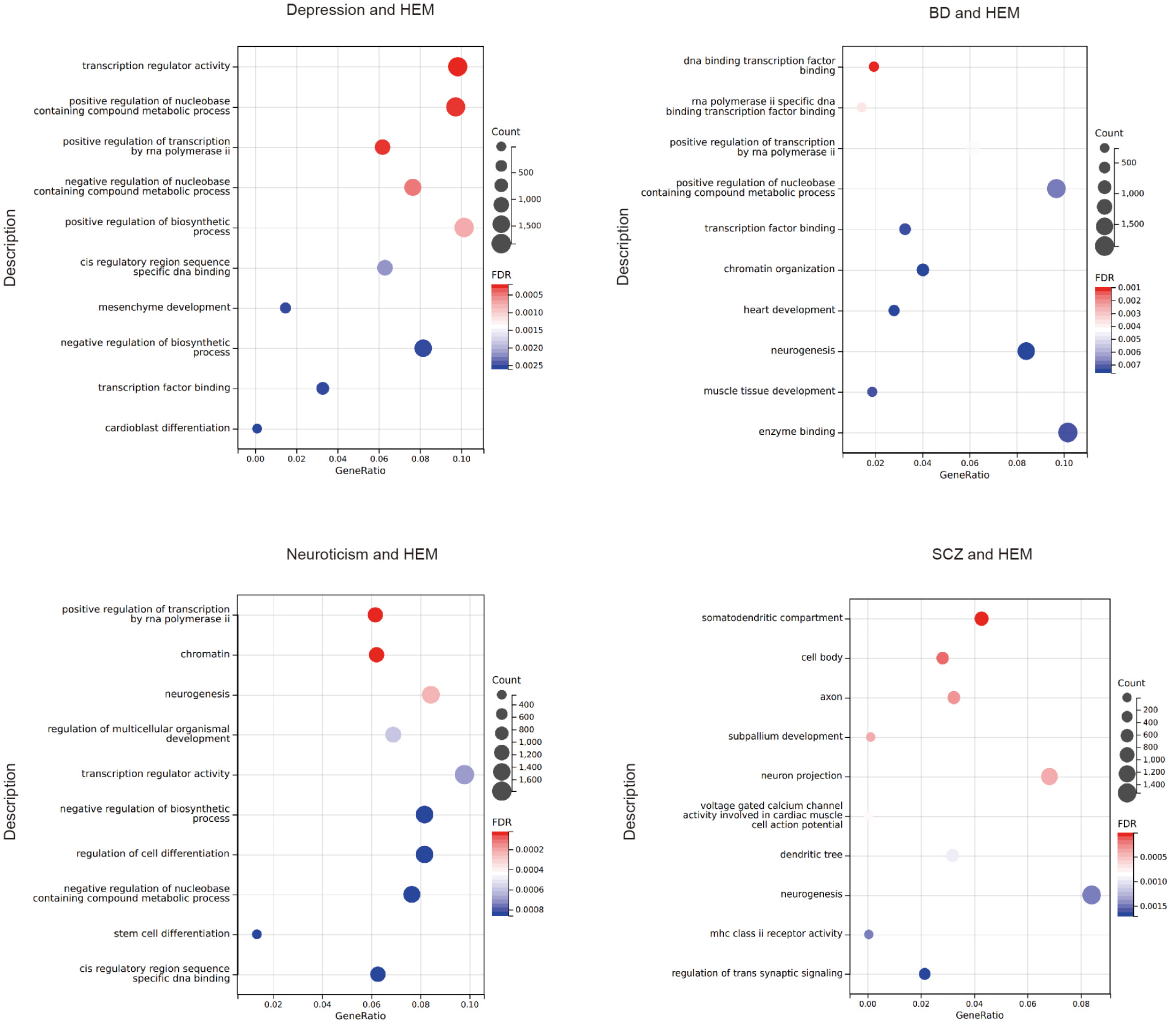
The bubble plot of the GO analysis. HEM, hemorrhoidal disease; BD, bipolar disorder; SCZ, schizophrenia.

GO analysis revealed that the target genes associated with psychiatric disorders significantly enriched in biological processes such as positive regulation of the nucleobase-containing compound metabolic process, positive regulation of transcription by RNA polymerase ii, neurogenesis, stem cell differentiation, and muscle organ development. The molecular functions focused on transcription factor binding, specific DNA binding, nuclear receptor binding, and calcium channel activity (**Figure 4**; **Supplementary Table S21**). The KEGG analysis of SCZ indicates that pleiotropic genes are enriched in the MAPK signaling pathway. However, no significant KEGG analysis after FDR correction was found for the other pairwise traits.

### 3.6 Mendelian randomization analysis

Finally, we assessed the causal relationship between psychiatric disorders and HEM using a bidirectional two-sample MR analysis. There were 40, 52, 90, and 150 instrumental variables for depression, BD, neuroticism, and SCZ, respectively, that reached genome-wide significance. Each SNP had an F-value greater than 10 (**Supplementary Table S22**). Random effects IVW analysis indicated significant positive associations between depression (OR = 1.15, 95% CI = 1.08-1.22, P = 1.72E-05), neuroticism (OR = 1.16, 95% CI = 1.06-1.27, P = 9.21E-04) and SCZ (OR = 1.03, 95% CI = 1.01-1.04, P = 4.34E-04) and increased HEM risk (**Supplementary Table S23**, **Supplementary Figure S3, S4**). The results of MRlap indicate that MR outcomes are affected by sample overlap. However, by correcting for sample overlap using MRlap, the causal effect of psychiatric disorders on HEM determined by MRlap is consistent with the primary MR analysis’s causal effect, ensuring the robustness of the IVW method (**Supplementary Table S25**). Despite heterogeneity, the MR-Egger intercept test indicates the absence of horizontal pleiotropy. The leave-one-out results confirmed the reliability of the overall causality (**Supplementary Figure S5**). However, the inverse MR analysis did not reveal a definitive causal relationship of genetic susceptibility between HEM and psychiatric disorders (**Supplementary Table S24**).

## 4 Discussion

To our knowledge, this study was the first comprehensive genome-wide cross-trait analysis of the shared genetic basis between common psychiatric disorders and HEM using large GWAS. Using genetic variation as an instrumental variable, we found some genetic associations between psychiatric disorders and HEM. First, we assessed the genetic correlations between depression and HEM, BD and HEM, neuroticism and HEM, and SCZ and HEM at both global and local levels. A significant positive correlation was found in all four pairs of traits. Secondly, our study identified multiple shared loci and colocalization evidence through pairwise analysis. Furthermore, we localized to 2304 genes and identified enriched biological processes. Subsequently, the cross-trait genes were validated at the transcriptome and proteome levels. Finally, the MR Analysis provided evidence for the causal relationship between depression, neuroticism, and SCZ and HEM.

We identified 17 pleiotropic loci between psychiatric disorders and HEM through cross-trait and colocalization analysis, obtaining multiple candidate genes. Multiple loci carried genes related to nervous system (MTCH2, MYT1L, CELF4, RSRC1, ZNF592, SHOX2) (37–41), cell proliferation and differentiation (CUL9) (42), and lipid transport (OSBPL2) (36). MYT1L, CELF4 and MTCH2 were given priority as novel pleiotropic genes between depression and HEM. MYT1L is associated with neurons in the brain and encodes MYT1L protein, a member of a zinc finger superfamily of neuronal transcription (37). MYT1L plays an important role in neural development, and is involved in intercellular synaptic transmission, axon development, and neurite growth (43). The CUGBP Elav-like family (CELF) can regulate RNA stability and protein translation, affecting neural development and closely related to neurological diseases (39). CELF4 is significantly enriched in neurons and neuroblasts, mainly in peptidergic neurons. The mitochondrial carrier homolog 2 (MTCH2) was the most significant candidate gene between depression and HEM. As a mitochondrial outer membrane protein, MTCH2 can regulate mitochondrial metabolism and related cell death (44). Research has shown that mitochondrial dysfunction is significantly associated with severe depression (MDD) (45). Kuffner et al. found that fibroblasts from patients with depression had significantly impaired mitochondrial function, as indicated by decreased respiration and decreased adenosine triphosphate (ATP) -related oxygen consumption (46). Single-cell transcriptomics studies indicated that fibroblasts played a critical role in the development, stability, and disease processes of the intestine (47). Our study also found that pleiotropic SNPS between depression and HEM were significantly enriched in fibroblasts (**Supplementary Figure S2**). Additionally, the effects of MTCH2 on neurological diseases and adipocyte differentiation have been increasingly recognized in recent years. The study by Cristen et al. pointed to the importance of MTCH2 as a highly expressed gene in the central nervous system in obesity susceptibility (48). Obesity-related oxidative stress tends to promote mitochondrial dysfunction and DNA damage (49). In summary, MTCH2 may be a common risk gene for psychiatric disorders and HEM, highlighting the role of mitochondrial dysfunction in the nervous system.

CUL9 is a member of the Cullin protein family, which plays a crucial role in regulating DNA damage response, cell proliferation, and apoptosis (42). Additionally, CUL9 is involved in the ubiquitination process of various substrates associated with cellular functions (50, 51). The polymorphism of RSRC1 and the brain functional changes in SCZ have been reported in previous studies (52). The mutations of RSRC1 triggered the decay of RSRC1 transcript mRNA in the fibroblasts of patients. ESR1 encodes estrogen receptor α (ER). Estrogen regulates mood-related neurotransmission through receptor-mediated and involved in the interaction of the hypothalamic-pituitary-adrenal axis (53). Plec-encoded lectin located in the chr8:144973183-145086428 (index SNP rs55646585) is a large cell junction protein widely distributed in many tissues, which is highly expressed in skin, muscle, and brain (54). Lectins promote cell-to-cell adhesion. In the brain, PLEC is co-localized with glial fibrillary acidic protein and tau protein to help the structural integrity of astrocytes and neurons (54). In a recent study, PLEC alterations were found to impair the adhesion of EBS-MD fibroblasts (55). The occurrence of muscle malnutrition can cause the intestinal mucosa and muscles to become weak, leading to the downward migration of the mucosa (56). Likewise, further studies are needed to elucidate the complex underlying biological mechanisms.

We obtained evidence of other genes through the results of TWAS and PWAS. PTK7, a pseudo tyrosine kinase lacking catalytic activity, is involved in the occurrence and development of a variety of cancers and is associated with cell survival, growth, and migration (57). Animal experiments showed that PTK7 cleavage in enteroendocrine cells activated the non-canonical Wnt signaling pathway in intestinal stem cells and promoted the migration of stem cells to the wound (58). Two roles of PTK7/Otk in patterning and neurogenesis were observed in a recent study, with OTk-1 expression observed in epithelial and neuronal cells during embryogenesis (59). This may suggest a role for PTK7 in neuronal cell migration. ITIH family genes are common genetic risk factors for a variety of psychiatric disorders (60, 61). The regulatory variant rs2535629 in the ITIH3 intron contributes to SCZ risk (62). ITIH4 was identified as a gene expressed by vascular smooth muscle cells in atherosclerotic plaques and was also associated with inflammatory responses (63).

Additionally, shared genes are enriched in various brain tissues. The shared genes of all pairwise traits showed significant enrichment in the cerebellum, which expressed that the occurrence of comorbidity may mainly depend on abnormal neurological dysfunction. In recent years, the role of the cerebellum in non-motor functions such as cognition and emotion has gradually been understood (64). Studies have shown that changes in the morphology or connectivity of the cerebellum can be observed in dominant or threatened SCZ and BD (65). Another study found that patients with SCZ, BD, or severe depression had altered expression levels of multiple GABAA receptor proteins in the cerebellum lateral region, which helps explain the similarity in the underlying basis of these diseases (66). The enrichment of functions and pathways indicated that pleiotropic genes affect the transcription and expression of downstream genes by regulating the binding process of transcription factors to DNA. It is well known that even within the same organism, the same transcription factors can recognize and control different genes through different assembly modes (67), and are widely involved in embryonic development and cell differentiation (68). Pleiotropy of transcription factors explained the identified targets of comorbidity may play important roles in two different diseases. This is validated by biological process enrichment, where mutations in shared genes may already affect our health during development.

Finally, the MR analysis provided evidence of a causal effect of the three psychiatric disorders on HEM. Consistent with previous findings, genetic susceptibility to HEM was significantly associated with the risk of depression. Our MR results extend the existing MR analysis. For depression, we used a larger sample size for GWAS and explained the potential sample overlap in pairwise traits using MRlap. Future studies with larger sample sizes or more observational studies are needed to complement the work on genetic information.

Our study has several limitations. First, we only focused on large European datasets to reduce population stratification. More data sets on psychiatric disorders and HEM in other populations are needed to generalize our conclusions. Second, there was a certain degree of sample overlap in the selected GWAS dataset. However, the bias introduced by overlapping samples was small, and the study was not affected by this. Finally, we need more experimental studies to validate the potential mechanisms in our research.

## 5 Conclusion

This study revealed a genetic correlation between four psychiatric disorders and HEM, identified 17 multi-effective genetic loci and multiple candidate genes, and confirmed causal relationships. This has facilitated our understanding of the common genetic mechanism, providing new targets for the treatment of patients with dual chronic burdens of psychiatric disorders and HEM.

## Data Availability

The original contributions presented in the study are included in the article/**Supplementary Material**. Further inquiries can be directed to the corresponding author.
