# Supplementary material for: Shared genetic architecture of psychiatric disorders and hemorrhoidal disease: a large-scale genome-wide cross-trait analysis
Source: Front Psychiatry. 2024 Nov 11;15:1456182. doi: 10.3389/fpsyt.2024.1456182 (PMC11586368; doi:10.3389/fpsyt.2024.1456182)
Supplement: Supplementary file 1 [file Image1.pdf]

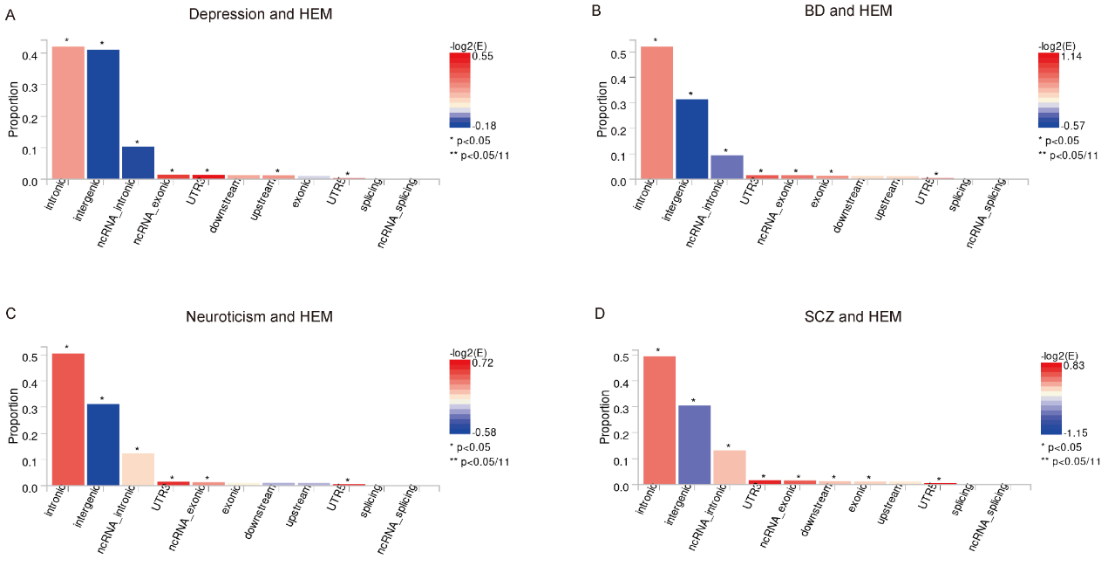

Supplementary figure 1. The proportion of 210 SNP category annotation. HEM: hemorrhoidal disease, BD: bipolar disorder, SCZ: schizophrenia.

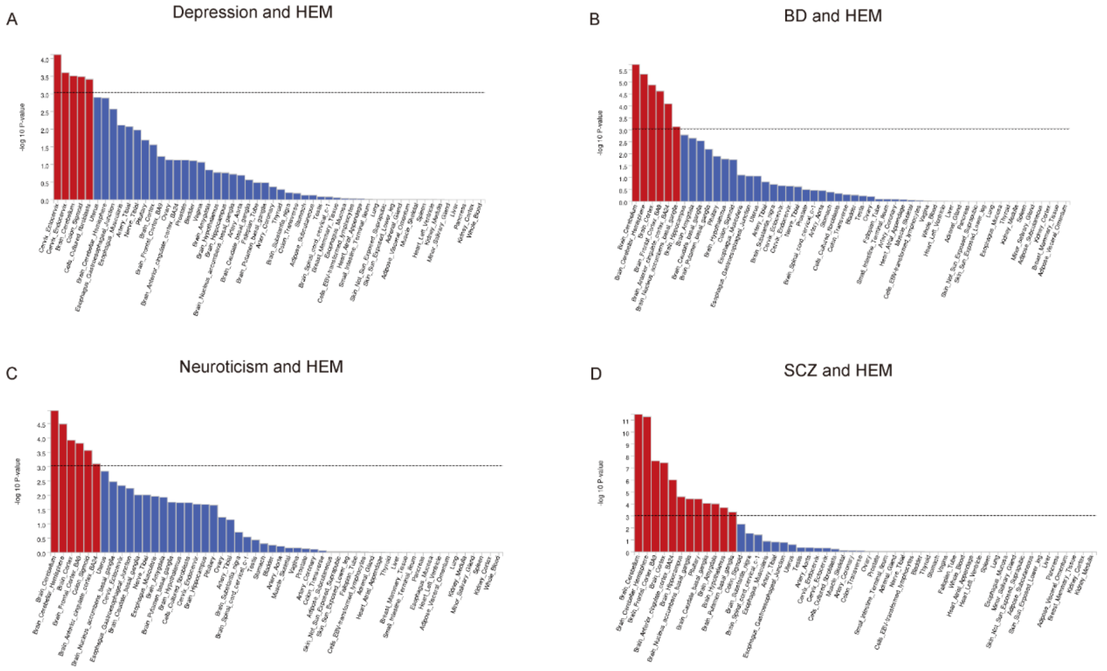

Supplementary figure 2. Results of GTEx 8 tissue enrichment. Red represents significant tissue and blue represents tissue that did not reach the threshold.

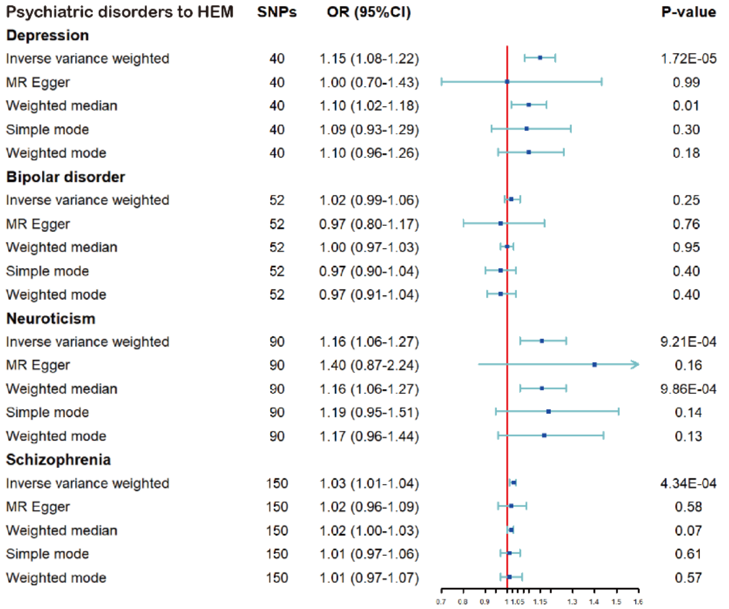

Supplementary figure 3. Forest plots to visualize causal effect of psychiatric disorders on HEM.

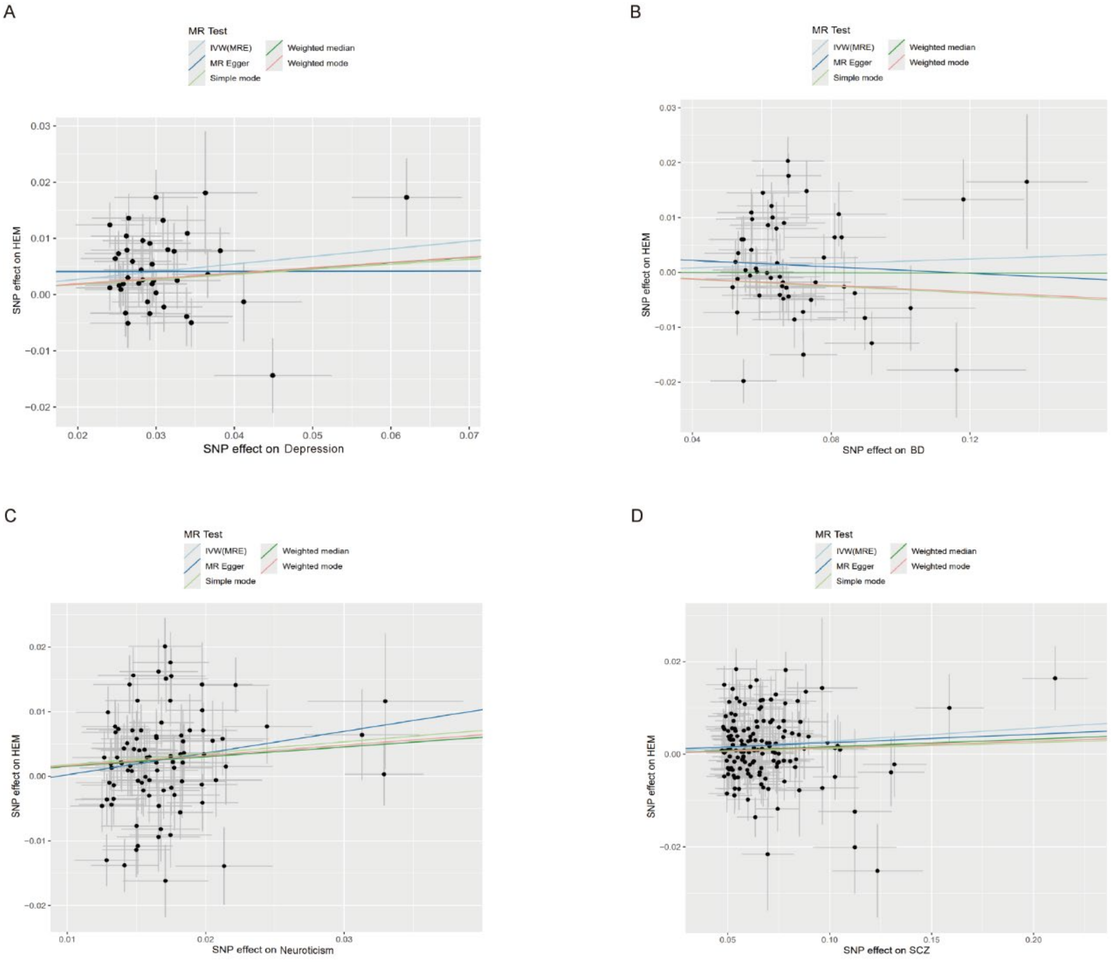

Supplementary figure 4. Scatter plots for the causal association between psychiatric disorders and HEM.

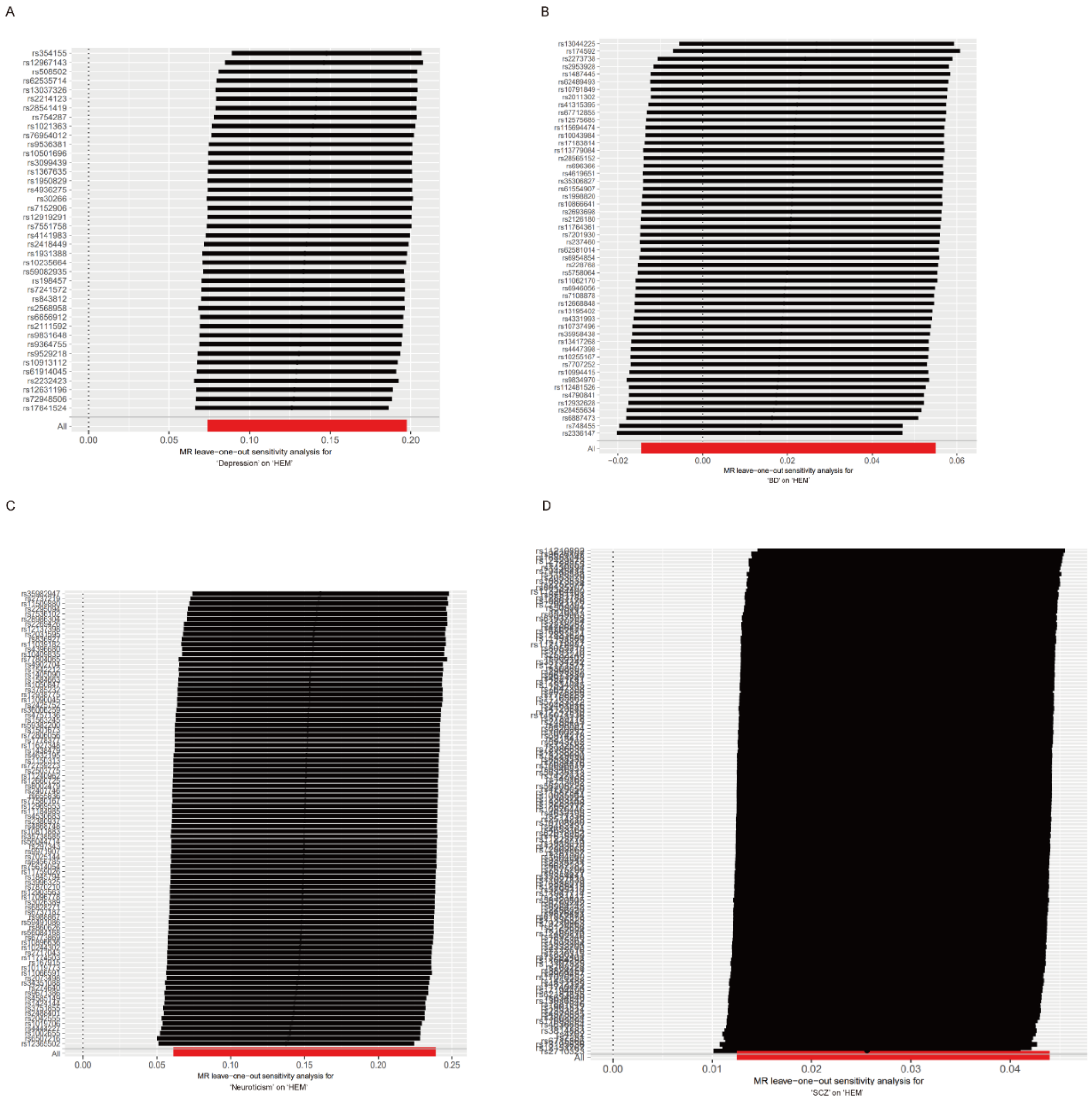

Supplementary figure 5. The forest plots of leave-one-out analysis between psychiatric disorders and HEM.
